# Enhancing the Plasma-Resistance Properties of Li_2_O–Al_2_O_3_–SiO_2_ Glasses for the Semiconductor Etch Process via Alkaline Earth Oxide Incorporation

**DOI:** 10.3390/ma16145112

**Published:** 2023-07-20

**Authors:** So-Won Kim, Hwan-Seok Lee, Deok-Sung Jun, Seong-Eui Lee, Joung-Ho Lee, Hee-Chul Lee

**Affiliations:** 1 Department of Advanced Materials Engineering, Tech University of Korea, Siheung 15073, Republic of Korea; swkim9193@tukorea.ac.kr (S.-W.K.); jds2697@tukorea.ac.kr (D.-S.J.); selee@tukorea.ac.kr (S.-E.L.); 2 Korea Evaluation Institute of Industrial Technology, Seoul 06152, Republic of Korea

**Keywords:** plasma resistant glass, LAS glass, alkaline earth oxide, low dielectric constant, low coefficient of thermal expansion, nonvolatile fluoride, semiconductor etch process

## Abstract

To develop plasma-resistant glass materials suitable for semiconductor etching processes, we introduced alkaline earth oxides (ROs) into a Li_2_O–Al_2_O_3_–SiO_2_ (LAS) glass. Analysis of glass properties with respect to the additives revealed that among the analyzed materials, the LAS material in which Li_2_O was partially replaced by MgO (MLAS) exhibited the most favorable characteristics, including a low dielectric constant (6.3) and thermal expansion coefficient (2.302 × 10^−6^/°C). The high performance of MLAS is attributed to the high ionic field strength of Mg^2+^ ions, which restricts the movement of Li^+^ ions under the influence of electric fields and thermal vibrations at elevated temperatures. When exposed to CF_4_/O_2_/Ar plasma, the etching speed of RO-doped glasses decreased compared with that of quartz and LAS glass, primarily owing to the generation of a high-sublimation-point fluoride layer on the surface. Herein, MLAS demonstrated the slowest etching speed, indicating exceptional plasma resistance. X-ray photoelectron spectroscopy analysis conducted immediately after plasma etching revealed that the oxidation-to-fluorination ratio of Li was the lowest for MLAS. This observation suggests that the presence of Mg^2+^ ions in the plasma discharge inhibits the migration of Li^+^ ions toward the surface, thereby contributing to the excellent plasma resistance of MLAS.

## 1. Introduction

The ever-growing demand for high-performance and highly integrated semiconductor products in the modern semiconductor industry necessitates the development of new manufacturing technologies and processes. Among these, micro-patterning using extreme ultraviolet (EUV) has emerged as an innovative technique in semiconductor manufacturing. The formation of fine patterns in the nanometer range through semiconductor etching requires high-density plasma to achieve precise, high aspect ratio patterns [1,2,3,4]. With the employment of high-density plasma, the chamber environment in semiconductor etching equipment has become increasingly harsh. Consequently, the poor durability of ceramic components in the chamber during plasma etching has resulted in device defects caused by contaminant particles, leading to reduced productivity [5,6,7,8].

Previous reports have highlighted that highly corrosion-resistant ceramic materials such as Y_2_O_3_, Al_2_O_3_, and quartz, which are currently used in semiconductor process chambers, can introduce contaminant particles due to surface defects and shorten product lifespans through local etching [9,10,11,12]. Consequently, extensive research has been conducted on alternative materials known as plasma-resistant glasses (PRGs) to replace these ceramic materials [8,13,14,15]. Plasma-resistant glasses exhibit superior resistance to plasma compared to conventional ceramics due to the high sublimation temperature of fluorides generated on the glass surface when exposed to plasma. As a result, various additives capable of forming fluorides with high sublimation points have been investigated [16,17]. Recently, plasma-resistant glasses based on alkaline earth oxide (RO)–Al_2_O_3_–SiO_2_ (RO: alkaline earth oxide) compositions have gained attention due to their cost-effectiveness and ease of glass formation compared to rare-earth-based glasses. Notably, Choi et al. reported excellent plasma resistance in CaO–Al_2_O_3_–SiO_2_ (CAS) glasses. However, these glasses have limitations such as high thermal expansion coefficients and vulnerability to thermal shock damage [18,19]. In the etching process, the electric field of plasma-resistant glasses can become concentrated or dispersed in specific regions due to plasma disturbance and non-uniform plasma formation in the chamber when the dielectric constant increases. Therefore, the development of low dielectric constant materials is crucial to ensure uniform etching depths and geometries across different regions, preserving etching uniformity [20,21,22]. 

The objective of this study was to develop a ceramic material with excellent plasma resistance and a low dielectric constant for application in semiconductor etching equipment. Initially, Li_2_O–Al_2_O_3_–SiO_2_ (LAS) glass, known for its low thermal expansion properties, served as the base component, with group 2 oxides, RO (R: Mg, Ca, Sr, and Ba), added to enhance etching resistance. The study investigated the effects of RO additions on the thermal and electrical properties of RLAS plasma-resistant glasses. Furthermore, we examined the plasma etching mechanism of RLAS glasses prepared through RO additions and explored the factors influencing changes in plasma resistance properties.

## 2. Materials and Methods

### 2.1. Glass Sample Fabrication Method

The LAS glass used in this study was designed with the basic composition of 15Li_2_O–20Al_2_O_3_–64SiO_2_ (mol%); this was performed by selecting a composition within the vitrification range in the LAS phase diagram, and the glass was prepared through the melting-quenching method. Li_2_O, Al_2_O_3_, and SiO_2_ powders (≥99%, Kojundo Chemical Laboratory, Saitama, Japan) were used as the starting materials for the glasses; the composition is listed in Table 1. The RLAS materials in which Li_2_O was partially replaced by MgO, CaO, SrO, and BaO (≥99%, Kojundo Chemical Laboratory, Saitama, Japan) in the composition were denoted as MLAS, CLAS, SLAS, and BLAS, respectively. The fining agents As_2_O_3_ and SnO_2_ were added at 0.5 mol% each to suppress the formation of pores in the glass. To fabricate glass samples, the weighed powders were mixed uniformly with ethanol and zirconia balls using a ball mill for 24 h. Subsequently, the mixture was dried at a temperature of 120 °C for approximately 6 h or more. The mixture was then placed in an alumina crucible and heated to 1650 °C for approximately 2 h to melt the glass. The melt was molded by pouring it into a graphite mold and then annealed at a temperature of about 50 °C above the glass transition temperature (Tg) for 3 h to eliminate the stress in the glass. The glass sample was then fabricated using a diamond blade with a thickness of about 0.25 µm to obtain a volume of 20 × 20 × 3 (mm^3^) before being polished. 

### 2.2. Evaluation of Glass Properties

To identify the crystalline phase of the glass melt, the glass samples were ground to powder using a planetary mixer (PULVERISETTE 7, Fritsch, Idar-Oberstein, Rhineland-Palatinate, Germany) and subjected to X-ray diffraction (XRD, D2 PHASER, BRUKER, Billerica, MA, USA) analysis. For glass transition temperature and glass crystallization temperature measurements, differential thermal analysis (DTA; STA449 F3, NETZSCH, Bavaria, Germany) was conducted to measure them in the temperature range from 25 to 1200 °C under an N_2_ atmosphere at a heating rate of 10 °C/min. In addition, the coefficient of thermal expansion was measured in the temperature range from 30 to 500 °C at a heating rate of 10 °C/min in the atmosphere using a thermomechanical analyzer (TMA, TMA 402 F3, NETZSCH, Bavaria, Germany). The density of each fabricated glass sample was obtained using distilled water as a fluid by the Archimedes method, and the value of C_p_, the capacitance, was measured at a frequency of 1 MHz using an LCR meter (E4980, Agilent, Santa Clara, CA, USA). Subsequently, the dielectric constant (*ε*_0_) of each glass sample was calculated by substituting the sample area (*A*), sample thickness (*t*), and the permittivity of vacuum (*ε_r_*) into Equation (1).

(1) $$\varepsilon_{r}=\frac{C_{p}\times t}{\varepsilon_{0}\times A}$$

To evaluate the plasma resistance, the processed glass samples were etched using capacitive coupling plasma-reactive ion etching (CCP-RIE) equipment according to the process conditions listed in Table 2. Before etching, the glass samples were prepared by cleaning them in an ultrasonic cleaner with acetone, ethanol, and IPA for 10 min each at 50 °C for a total of 30 min. Plasma etching was performed for 2 h with a total of 12 cycles of etching for 10 min followed by 1 cycle of rest for 10 min to prevent excessive etching due to plasma heating. Etching was performed by changing the position of the glass sample every 2 cycles. To compare the etch rate of each sample, the weight of the sample before and after etching was measured using an electronic balance with a precision of 10^−5^ g to obtain the weight etched per hour (ΔW). The etch rate was then calculated by substituting the density (*ρ*) and area (*A*) of the sample into Equation (2).

(2) $$Etch\;rate=\frac{∆W}{\rho \times A}$$

## 3. Results and Discussion

**Figure 1:**
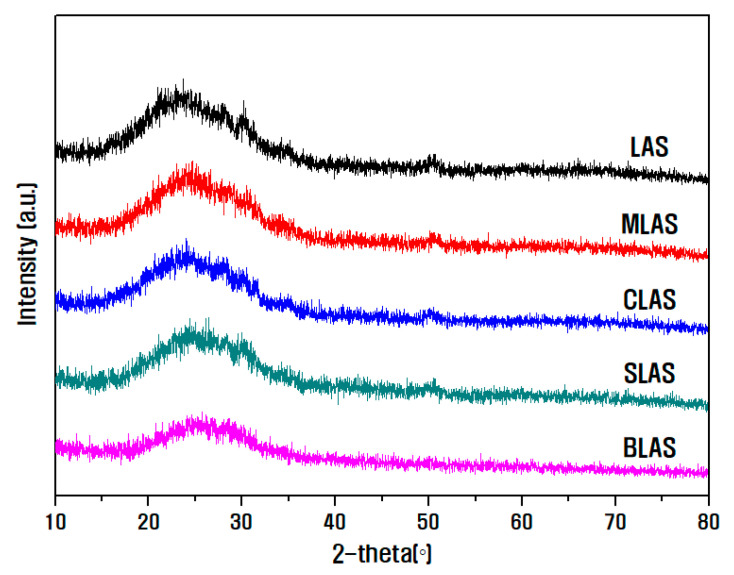
XRD pattern analysis results of the produced LAS and RLAS glass materials.

Figure 1 presents the analysis results of the XRD pattern of the LAS and RLAS glass powders produced in this study. All glass compositions exhibited a typical amorphous diffraction pattern with a gentle hill shape at approximately 20°−30°, whereas the crystalline phase was barely observed.

Table 3 lists the various properties of the added elements that comprise the RLAS glass to determine the cause of the changes in the properties of the glass samples due to the added element. The ionic field strength (F) represents the force acting on oxygen at a distance of r_c_ (r_c_ = r_i_ + r_0_, r_i_ is the cation ionic radius and r_0_ is the oxygen ionic radius) from the cation of each added element. It was determined using the equation F = Z_i_/r_c_^2^ (Z_i_ is the ionic charge), and the various properties of each element were predicted to contribute to the changes in the glass properties [23,24]. 

**Figure 2:**
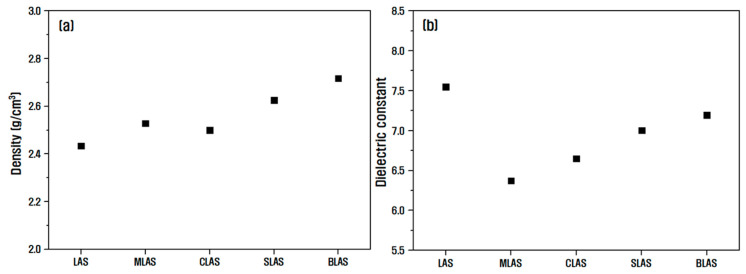
Measurement results of (**a**) density and (**b**) dielectric constant of LAS and RLAS glass samples.

Figure 2 shows the measurement results of the density and dielectric constant of LAS and RLAS glass samples. The density of each glass sample in Figure 2a indicates that the density generally increases with the atomic weight of the added element. However, in the MLAS glass samples, Mg had a smaller atomic weight than Ca but a slightly higher density. This is considered to be due to the overall decrease in volume when Mg was added, which increased the density. There are three possible reasons for this outcome. First, the Mg^2+^ ions have a small ionic radius (r_Mg_ is 0.078 nm), and this reduces the physical lattice constant. Second, alkaline earth oxides in the network consisting of covalent bonds of tetrahedral Al–Si–O form ionic bonds with oxygen ions when introduced as cations. However, the Mg^2+^ ions exhibit a high ionic strength with oxygen and react closer to covalent bonds than other divalent elements, resulting in a shorter bond length. Finally, Mg^2+^ ions participated in the formation of the network, and this is thought to have caused the volume reduction because they have a similar atomic radius to Al acting as an intermediate oxide in the glass, which facilitated substitution in the network [25,26,27]. Figure 2b shows the dielectric constant measured at 1 MHz for each glass sample. When a high-frequency alternating electric field was applied to the LAS glass, ion polarization was the main mechanism, causing the ions in the network to react and migrate. Thus, it was expected that Li^+^ ions, which have the smallest ionic radius and atomic weight among the added elements, would dominate the dielectric constant properties due to their small size and light weight, resulting in high mobility [28,29,30]. Therefore, the LAS sample with the largest Li content among the glass samples exhibited the largest dielectric constant of 7.5. The dielectric constants of RLAS samples with alkaline earth oxides increased in the order of MLAS < CLAS < SLAS < BLAS. This is thought to be influenced by the ionic radius and ionic field strength of the elements. Amma et al. reported that when ions of different sizes are mixed, the dielectric constant increases with the ionic radius of the largest ion, and the voids in the network formed by increasing the ionic radius of the largest ion can serve as a pathway for the movement of smaller ions [31]. Therefore, the smaller ionic radius of the element added to the RLAS sample is presumed to have formed the smallest voids in the network, which hindered the movement of Li^+^ ions, resulting in a lower dielectric constant. Furthermore, when Li^+^ ions are moved by an external electric field, collisions with cations exert a large repulsive force proportional to the ionic field strength, which reduces the mobility of Li^+^. Hence, the MLAS sample with Mg added, which has the smallest ionic radius (r_Mg_ is 0.078 nm) and the largest ionic field strength (F_Mg_ is 385.8 nm^−2^), had the lowest dielectric constant of 6.3.

**Figure 3:**
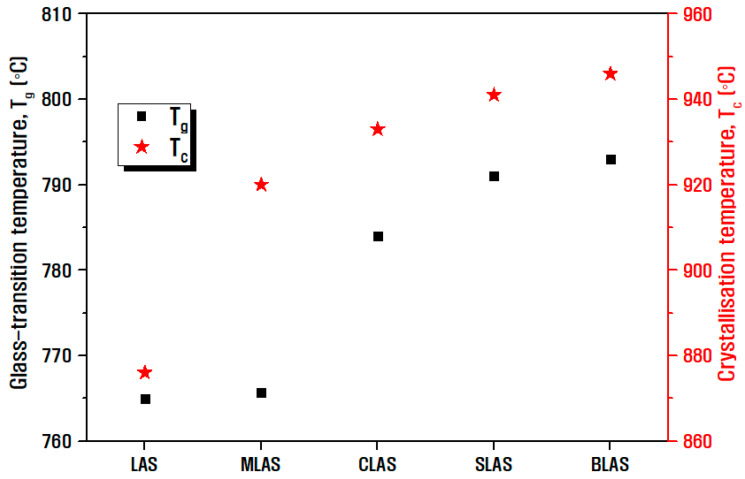
Measurement results of glass transition temperature (T_g_) and crystallization temperature (T_c_) of LAS and RLAS glass samples.

**Figure 4:**
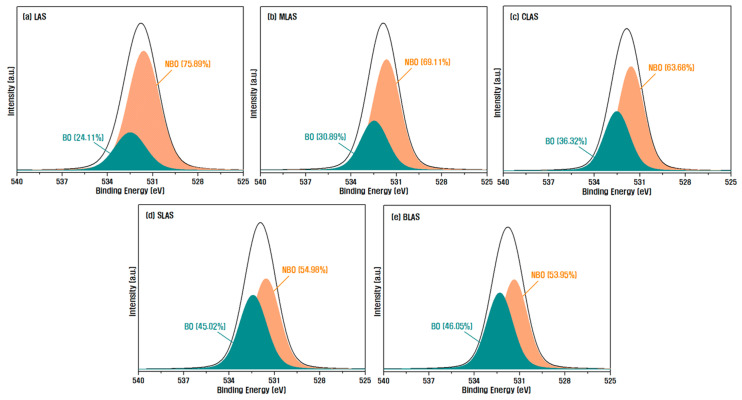
XPS narrow scan analysis results for O1s of each glass sample.

Figure 3 presents the analysis results of the glass transition temperature (T_g_) and crystallization temperature (T_c_) through the heat flow of DTA as a function of temperature. The aluminosilicate glass structure is based on the (Si,Al)O_4_ tetrahedron, where the Al and Si are strongly covalently linked to four bridging oxygen atoms. When an alkali oxide or alkaline earth oxide is added to this tetrahedral structure as a network modifier, it ionically bonds with oxygen to form non-bridging oxygen (NBO). Perturbation occurs when the NBO that forms the network increases, indicating that the bond structure of the glass is weakened. At this point, as the ionic field strength of the cations that are network modifiers increases, the perturbation also increases. This is known to lower the glass transition temperature and increase the crystallization tendency of the glass, resulting in a lower crystallization temperature [19,32,33,34]. Therefore, as shown in Figure 4, which shows the area ratio of NBO and BO by separating the O1s peak obtained through XPS, small and light Li^+^ ions exhibited the lowest glass transition temperature and crystallization temperature due to their high mobility and ability to move quickly within the glass network and ionically combine with covalently bound oxygen to form NBO. This behavior surpasses the performance of alkaline earth oxides. In the case of the glass samples with RO added to the LAS glass, as the ion field strength of the group 2 elements shown in Table 3 decreases, NBO decreases, and the degree of network connectivity increases, confirming that the glass transition temperature increases. The MLAS glass sample with Mg addition had a large ionic field strength and showed an insignificant temperature increase compared to the LAS sample. In addition, the thermal stability of a glass against crystallization can be estimated using the common stability criterion represented by ∆T = T_c_ − T_g_. The higher value of ∆T indicates higher thermal stability and glass-forming ability. The value of ∆T was 111 °C in the LAS composition, whereas this value increased to 150–155 °C when RO was incorporated, confirming that the addition of RO can improve the thermal stability of glass [35,36,37].

**Figure 5:**
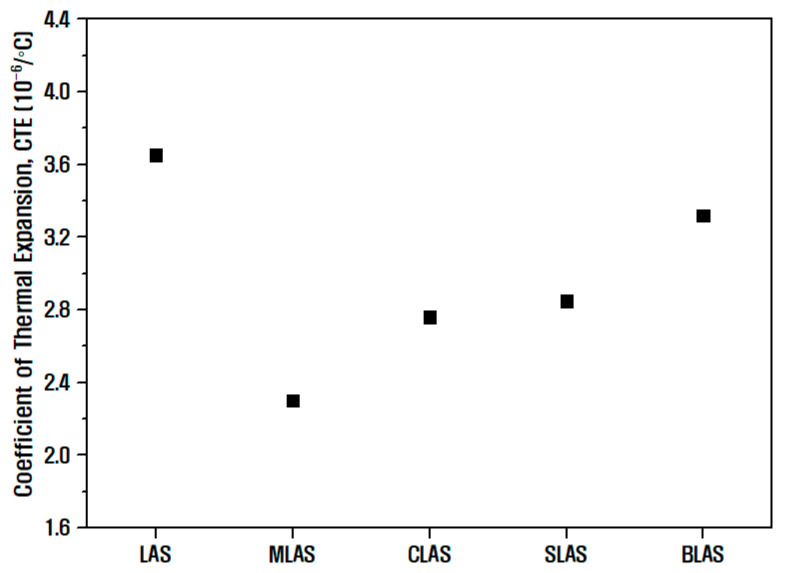
Measurement results of the thermal expansion coefficient of LAS and RLAS glass samples.

Figure 5 shows the coefficient of thermal expansion for LAS and RLAS. Thermal expansion refers to the expansion of the material due to increased vibrational amplitude and potential energy asymmetry between two atoms at rising temperatures. Hence, the coefficient of thermal expansion increases as the asymmetry increases [38,39,40,41]. If the ionic strength of the added element is high, it generally forms a stronger bonding structure, which reduces the thermal expansion coefficient because the amplitude of the thermal vibration in the network is not large. Thus, in the RLAS glass sample with alkaline earth oxides, the coefficient of thermal expansion changes inversely proportional to the ionic strength of the binary element shown in Table 3. The MLAS sample mixed with Mg^2+^ ions, which have the largest ionic field strength, exhibited the lowest coefficient of thermal expansion of 2.302 × 10^−6^/°C.

**Figure 6:**
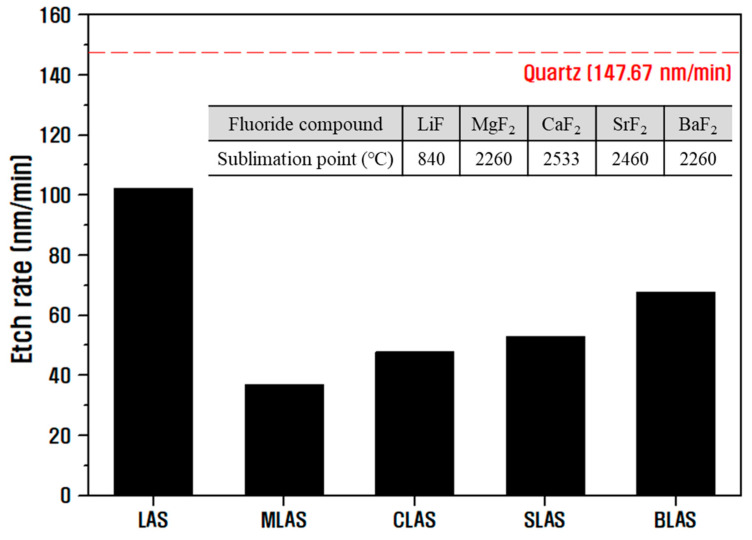
Etch rate measurement results for fabricated LAS and RLAS glasses versus quartz (inner table: comparison of sublimation points of fluorides involved).

Figure 6 shows the change in etch rate versus quartz for LAS and RLAS glasses exposed to the CF_4_/O_2_/Ar plasma used in this study. All RLAS samples exhibited much lower etch rates than quartz, indicating excellent plasma resistance properties. When various alkali metals and alkaline earth metals are added to LAS glass, non-volatile fluorides with high sublimation points are formed on the surface due to the reaction with fluorine radicals in CF_4_ plasma. The sublimation points of the fluorides that can be formed in this study are summarized in Figure 6. Notably, the etch rate decreases because these fluorides do not volatilize and act as a barrier on the surface against ion bombardment [18,42,43]. Consequently, plasma corrosion etchability is presumed to be proportional to the sublimation point of the fluoride formed on the surface. Because LAS can only form LiF with a low sublimation point of 840 °C, it exhibits a relatively higher etch rate than other RLAS-based glasses. However, MLAS exhibited lower etch rates compared to CLAS samples, which form fluorides with the highest sublimation point. When glass samples are exposed to RF-powered plasma, ion polarization occurs within the glass, causing ions in the network to migrate toward the surface, where the formation of LiF will occur first due to the Li^+^ ions having the highest mobility. Presumably, this migration of Li^+^ ions generates a negative space charge in the network, making it difficult for the slower inner alkaline earth metal ions to move to the surface to compensate for the charge [8,29]. We believe that the addition of Mg^2+^ ions with a small ionic radius and large ionic field strength most significantly hindered the movement of Li^+^, thereby facilitating the formation of alkaline earth fluoride on the surface of MLAS glass compared to other samples, resulting in the lowest etching rate. In contrast, the BLAS sample with Ba^2+^, which has the largest ionic radius and smallest ionic field strength, experienced the most difficulty in forming BaF_2_ on the surface due to its low mobility, resulting in a higher etch rate compared to the other RLAS glass samples. To examine the reasons for these plasma-resistant properties, the surface of each sample was analyzed via XPS (K-alpha+, Thermo Scientific, Waltham, MA, USA) immediately after the plasma etching process to determine the chemical bonding of the elements. 

**Figure 7:**
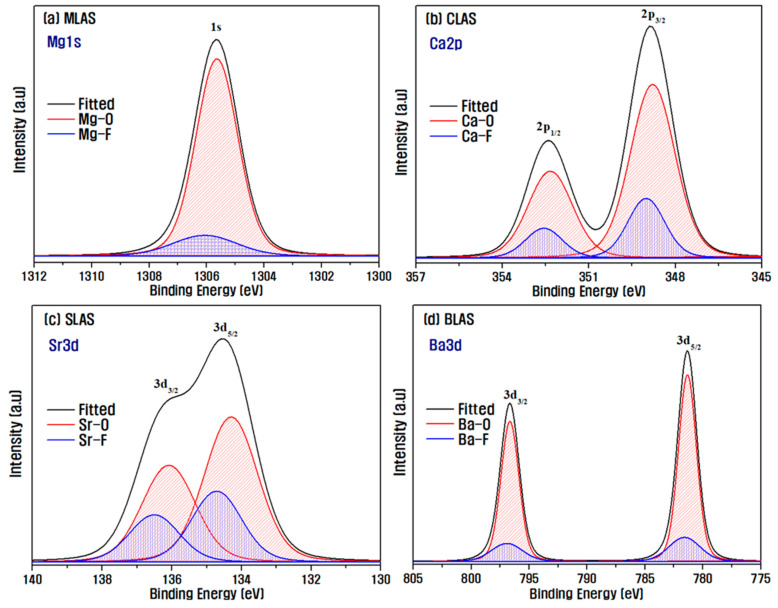
XPS narrow scan analysis results for alkaline earth metals on the surface of each RLAS sample immediately after the plasma etching process.

Figure 7 shows the results of the XPS analysis of alkali earth metals on the surface of the RLAS sample immediately after the plasma etching process. Peak separation of the data obtained via XPS analysis was performed [44]. As the electronegativity of fluorine is higher than that of oxygen, fluoride has a higher binding energy than oxide. For both RLAS samples mixed with alkaline earth oxides in LAS-based glasses, it was found that alkaline earth metal ions form fluorides through F radical reactions when they reach the surface. The relative proportions of F and O bonds are also shown in Table 4. In all samples, the ratio of R–F/R–O is relatively higher than that of Si–F/Si–O or Al–F/Al–O. This result indicates that alkaline earth fluorides have a higher sublimation point compared to SiF_4_ (−86 °C) and AlF_3_ (1291 °C). Thus, they volatilize relatively less and retain more on the glass surface [13,16]. Thus, the addition of alkaline earth oxides to LAS-based glasses could further improve the plasma-resistant properties of the glasses through the formation of fluorides with higher sublimation points on the surface. 

**Figure 8:**
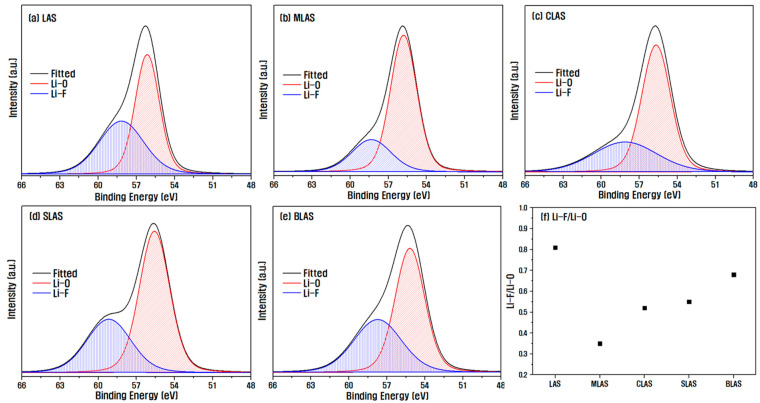
XPS narrow scan analysis results for Li on the surface of each sample immediately after the plasma etching process.

Figure 8 shows the results of XPS analysis of Li 1s on the sample surface to determine the extent of LiF formation on the surface depending on the added element when mixing alkali earth metals in LAS glasses. The Li 1s peaks were separated into Li–O and Li–F peaks to evaluate the relative amounts of oxidation and fluorination of Li on the surface. The change in the Li–F/Li–O ratio showed a trend almost similar to the etch rate in Figure 6. The MLAS sample showed the lowest ratio of 0.36, which is the lowest etch rate and thus the highest plasma resistance. As explained earlier, it is believed that the movement of Li^+^ ions to the surface was inhibited due to the influence of Mg^2+^ ions with large ion field strengths, and the formation of LiF with a low sublimation point was relatively difficult. 

Consequently, the study concludes that reducing the content of Li_2_O in LAS-based glasses and incorporating alkaline earth oxides, particularly Mg^2+^ ions with a small ionic radius and large ionic field strength, can lead to glass materials with exceptional plasma resistance. These glasses exhibit reduced Li^+^ ion mobility, resulting in lower dielectric constants, and preferentially form alkaline earth metal fluorides with high sublimation points on the surface.

## 4. Conclusions

This study aimed to analyze the thermal, electrical, and plasma resistance properties of LAS glasses with low thermal expansion. The evaluation involved the addition of binary oxides (RO) and explored the etching mechanism of RLAS glasses. The inclusion of RO had the effect of reducing the mobility of Li^+^ ions, thereby resulting in RLAS glasses with a lower dielectric constant compared to LAS glasses.

Specifically, the MLAS glasses incorporating the addition of MgO displayed a comparatively lower glass transition temperature (T_g_) of 765 °C, indicating their high meltability and excellent glass-forming capability. Additionally, these glasses demonstrated excellent thermal properties, as indicated by a thermal expansion coefficient of approximately 2.302 × 10^−6^/ °C. This impressive performance can be attributed to the hindrance caused by Mg^2+^ ions, which restricted the migration of Li^+^ ions to the greatest extent among the various ions. This hindrance was due to the combination of the low ionic radius and high ionic field strength of Mg^2+^ ions. Consequently, the surface of MLAS glasses facilitated the formation of alkaline earth metal fluoride, leading to the lowest etch rate and superior plasma resistance properties.

To enhance the thermal, electrical, and plasma-resistant properties of the glass materials, it is recommended to decrease the content of Li_2_O in LAS-based glasses and incorporate binary oxides, with a particular emphasis on MgO. These optimized glass compositions hold promise for utilization in semiconductor etching chambers that employ high-density plasma.

## Figures and Tables

**Table 1:** Composition of the (RO)–Li_2_O–Al_2_O_3_–SiO_2_ glass material used in this study.

Notation	Chemical Composition (mol%)								
	SiO_2_	Al_2_O_3_	Li_2_O	MgO	CaO	SrO	BaO	As_2_O_3_	SnO_2_
LAS	64	20	15	-	-	-	-	0.5	0.5
MLAS	64	20	7.5	7.5	-	-	-	0.5	0.5
CLAS	64	20	7.5	-	7.5	-	-	0.5	0.5
SLAS	64	20	7.5	-	-	7.5	-	0.5	0.5
BLAS	64	20	7.5	-	-	-	7.5	0.5	0.5

**Table 2:** Conditions for evaluating plasma resistance using CCP-RIE in this study.

Parameter	Condition
Frequency (MHz)	13.56
Power (W)	300
Pressure (mTorr)	10
Etch time (min)	60
CF_4_ (sccm)	30
O_2_ (sccm)	10
Ar (sccm)	5
Chiller Temp. (℃)	20

**Table 3:** Various properties of the added elements that comprise the RLAS glass used in this study.

	Si	Al	Li	Mg	Ca	Sr	Ba
Ionic Radius (nm)	0.039	0.057	0.078	0.078	0.106	0.127	0.217
Atomic Radius (nm)	0.117	0.143	0.152	0.160	0.197	0.215	0.217
Atomic Weight (amu)	28	27	7	24	40	88	137
Electronegativity	1.90	1.61	0.98	1.31	1	0.95	0.89
Ionic Field Strength (nm^−2^)	2500	1068	173.1	385.8	200	124	97.8

**Table 4:** Oxidation to fluoridation ratios of each element of the major cations comprising RLAS glasses immediately after plasma etching.

	R–F/R–O (R: Alkaline Earth)	Si–F/Si–O	Al–F/Al–O
MLAS	0.16	0.06	0.09
CLAS	0.28	0.05	0.07
SLAS	0.45	0.06	0.09
BLAS	0.20	0.08	0.12

## Data Availability

The data presented in this study are contained within the article.
